# Retinoic Acid Induced 1, *RAI1*: A Dosage Sensitive Gene Related to Neurobehavioral Alterations Including Autistic Behavior

**DOI:** 10.2174/138920210793360952

**Published:** 2010-12

**Authors:** Paulina Carmona-Mora, Katherina Walz

**Affiliations:** John P. Hussman Institute for Human Genomics, Dr. John T. Macdonald Foundation, Department of Human Genetics, Miller School of Medicine, University of Miami, Miami, Florida, USA

**Keywords:** Copy Number Variation, dosage sensitive gene, neurobehavioral traits, Potocki-Lupski Syndrome, RAI1, Smith-Magenis Syndrome, transcription factor activity.

## Abstract

Genomic structural changes, such as gene Copy Number Variations (CNVs) are extremely abundant in the human genome. An enormous effort is currently ongoing to recognize and catalogue human CNVs and their associations with abnormal phenotypic outcomes. Recently, several reports related neuropsychiatric diseases (i.e. autism spectrum disorders, schizophrenia, mental retardation, behavioral problems, epilepsy) with specific CNV. Moreover, for some conditions, both the deletion and duplication of the same genomic segment are related to the phenotype. Syndromes associated with CNVs (microdeletion and microduplication) have long been known to display specific neurobehavioral traits. It is important to note that not every gene is susceptible to gene dosage changes and there are only a few dosage sensitive genes. Smith-Magenis (SMS) and Potocki-Lupski (PTLS) syndromes are associated with a reciprocal microdeletion and microduplication within chromosome 17p11.2. in humans. The dosage sensitive gene responsible for most phenotypes in SMS has been identified: the Retinoic Acid Induced 1 (*RAI1*). Studies on mouse models and humans suggest that *RAI1* is likely the dosage sensitive gene responsible for clinical features in PTLS. In addition, the human *RAI1* gene has been implicated in several neurobehavioral traits as spinocerebellar ataxia (SCA2), schizophrenia and non syndromic autism. In this review we discuss the evidence of *RAI1* as a dosage sensitive gene, its relationship with different neurobehavioral traits, gene structure and mutations, and what is known about its molecular and cellular function, as a first step in the elucidation of the mechanisms that relate dosage sensitive genes with abnormal neurobehavioral outcomes.

## GENOMIC STRUCTURAL CHANGES AND NEUROPSYCHIATRIC TRAITS

Genomic structural changes, such as gene Copy Number Variations (CNVs) are extremely abundant in the human genome. Approximately 14,400 CNV loci larger than 1 kb were identified to date (http://projects.tcag.ca/variation/) [1]. In a recent article the impressive amount of more than 1,000 CNV/individual human genome ranging from 443 bp to 1.28 megabases (Mb), with a median size of 2.9 kb was reported [2]. On the other hand, an enormous effort is currently ongoing to recognize and catalogue human CNVs associated with abnormal phenotypic outcomes [3-7]. Some conditions affecting the central nervous system, such as schizophrenia, mental retardation (MR) and autism spectrum disorder (ASD) were shown recently to be associated with both the deletion and the duplication of the same genomic segment [8-13]. There is a great level of complexity between the presence of CNV and the resulting phenotypes that is not only a direct consequence of specific altered gene dosage. Analyses of the genome-wide functional impact of these structural variants showed that copy number changes not only cause alterations in expression levels of genes within CNVs but also influence the expression of genes in their vicinity [14, 15]. Moreover, a recent study clearly demonstrates that the presence of structural changes associated with CNV is enough to cause a phenotype independent of gene dosage [16]. 

Genomic disorders are the clinical manifestation of pathological CNV, they are frequent conditions (~ 1 per 1,000 births) and often sporadic resulting from *de novo* rearrangements [17]. In a subset of such conditions the rearrangements comprise multiple unrelated contiguous genes that are physically linked and thus have been referred to as Contiguous Gene Syndromes (CGS). An increasing number of CGS are being described each of them presenting a complex and specific phenotype. The beauty of these CGS is that there are specific phenotypes associated with certain genomic regions, thus making the detection of the genes involved in certain pathways easy. Although the segmental aneuploidy usually involves several genes, only a small subset of them conveys phenotypes as a function of copy number alteration. These particular genes are referred as “dosage sensitive genes”. However, the question of what makes a gene or its product a “dosage sensitive gene” remains to be elucidated.

Smith-Magenis (SMS, OMIM# 182290) and Potocki-Lupski (PTLS, OMIM# 610883) syndromes are two examples of CGS that are associated with a microdeletion and a microduplication respectively within chromosome 17 band p11.2. SMS was first described in 1986, and has a birth prevalence estimated at 1/15,000-1/25,000 [18-20]. The clinical phenotype includes craniofacial abnormalities, brachydactyly, self-injurious behavior, sleep abnormalities and mental retardation. Less commonly reported is cleft palate, congenital heart defects, seizures, hearing impairment and urinary tract anomalies. Molecular studies revealed a common deleted region of ~4 Mb in the majority of SMS patients (>70-80%) [21]. Unusual sized deletions (smaller or larger) were observed in 20–25% of patients. By examining the breakpoints in unusual sized deletions, the SMS critical region was redefined to a ~950 kb interval, in which fifteen genes and eight predicted genes were present [22] (Table **1**). PTLS was first described in 2000 [23] and is associated with the reciprocal duplication of the genomic interval deleted in SMS. The clinical presentation of PTLS includes mental retardation, autistic features, hyperactivity, developmental delay and dysmorphic features. In addition, PTLS patients present hypotonia, poor feeding and difficulty to thrive in infancy, oral-pharyngeal dysphasia, obstructive and central sleep apnea, structural cardiovascular abnormalities, electroencephalogram (EEG) abnormalities, behavioral abnormalities, and hypermetropia [24-26] (Table **1**).

## *RAI1* IS THE DOSAGE SENSITIVE GENE MOSTLY RESPONSIBLE FOR THE SMS AND PTLS CLINICAL PRESENTATION

As mentioned above, only a few genes do appear to be clearly haploinsufficient, such that hemizygotes exhibit a recognizable phenotype. Point mutations (nonsense and frameshift as well as missense alleles) in the Retinoic Acid Induced 1 gene (*RAI1*) were identified in patients with clinical presentation of SMS but no molecular deletion found by FISH [30-33] (Tables **1** and **2**), suggesting that *RAI1* is the dosage sensitive gene causative of SMS. In specific, there are two cases of patients with SMS that carry missense mutations that alter amino acids within a highly conserved region of the gene (A4685G and G5423A). Both patients have a wide spectrum of neurobehavioral phenotypes, including sleep disturbance, self-injurious behaviors, mental retardation and developmental delay. This supports the fact that RAI1 is a protein directly involved in these specific neurobehavioral phenotypes, since the point mutations mentioned above generate a full length protein with only the change of one single amino acid, with stability similar to the wild type protein [34].

In addition, a recent report showed a patient with PTLS phenotype having a duplication of a very small region that only contains the *RAI1 *gene [29]. This is consistent with the notion of *RAI1* as the dosage sensitive gene within this genomic region (Table **1**).

Additional support to the idea that *RAI1* is the dosage sensitive gene within this interval comes from studies in mouse models. Human chromosome 17p11.2 is syntenic to the 32-34 cM region of murine chromosome 11. The number and order of the genes are highly conserved [36-38]. By chromosome engineering, a strategy developed to introduce defined chromosomal rearrangements into the mouse genome to delineate gene haploinsufficiency effects (haploid genetics) [39-43], a deletion and reciprocal duplication in the mouse chromosome region syntenic to the SMS critical interval were generated [44]. The mouse model for SMS, *Df(11)17/+* or* Del,* carries a ~1.7 Mb deletion and presents craniofacial abnormalities, seizures, obesity, alterations in circadian rhythm and hypoactivity, decreased anxiety in the plus maze test, social interaction differences in the tube test and the social novelty test recapitulating most of the phenotypes observed in human patients [15, 44-47]. The mouse model for PTLS syndrome, *Dp(11)17/+* or *Dup*, carries a duplication of a region of ~1.7 Mb and are significantly leaner than wild type mice, hyperactive and exhibit impaired contextual fear conditioning, they display increased anxiety behavior in the plus maze test and increased dominance in the tube test. Abnormal sociability and preference for social novelty was also observed [15, 44, 45].

Interestingly enough, most of the phenotypes present in *Dup* mice were rescued in the *Dup/Rai1^-^* compound heterozygote (obtained by mating *Dup* mice with *Rai1^**+/- **^*mice, a model with *Rai1* haploinsufficiency) where the gene dosage of *Rai1* was 2n despite the presence of 3 copies of the other 23 genes in the region; being the first indication of *Rai1* as the dosage sensitive gene responsible the PTLS syndrome [50]. Consistent with this, there have been reports of mouse models with underexpression of Rai1 [48] and overexpression of Rai1 [51] that presented several of the expected phenotypes. For the last one, analyses in both hemizygous and homozygous transgenic mice revealed a dosage-dependent exacerbation of the phenotype, which included growth retardation, increased anxiety and hyperactivity [51]. A summary can be seen in Table **3**.

Utilizing the *Del* and *Dup* mouse models, the levels of expression of *Rai1* and the other genes within this specific genomic rearrangement were studied. Genome-wide expression levels in four organs (cerebellum, heart, kidney and testis) from adult male mice (at least three animals of *Dup*, *Del*, *Dup/Del* and wild type mice) showed a positive correlation between gene dosage and expression levels [15, 16]. The transcripts within the rearranged interval are expressed on average 0.66 (SD=0.15) fold less in the *Del* (one copy) and 1.38 (SD=0.29) fold more in *Dup *animals (three copies). They were, however, unchanged in *Del/Dup* mice (1.02 (SD=0.16) fold more, two copies in *cis*) compared to normal controls (two copies in *trans*) [16]. This clearly showed that *Rai1* gene copy number is correlated with levels of expression of the gene, and suggests that the protein levels are modified concomitant with gene dosage. Interestingly enough, most of the genes within this genomic interval behave in the same way, suggesting that is not the levels of  expression that make the difference between *Rai1* and the other 23 genes in the region. 

## MOLECULAR ASPECTS OF RAI1

*RAI1 *lies in the middle of the Smith-Magenis critical region, spanning over 120 kb. It consists of six exons, being the third exon the one that contains more than 90% of the coding region. 

As is shown in Fig. (**1**), there are several regions recognizable in RAI1 protein structure. A polymorphic CAG repeat encoding for a polyglutamine tract is present in the N-terminus of the RAI1 protein. Two polyserine tracts, each located at the N and C-terminal regions. The polyserine signal is similar to the one present in the *DRPLA* gene and the Drosophila hairless gene, both proteins involved in neuronal development [53, 54]. RAI1 has two putative bipartite nuclear localization signals (NLSs) *in silico* predicted. It also possesses a zinc finger like plant homeo domain (PHD) in the C-terminus, which is present in the trithorax family of chromatin remodeling transcriptional regulators [30, 31]. The isoform 1 of *RAI1* corresponds to the canonical sequence, and it is found in high levels mainly in brain and heart [55]. There are other three isoforms produced by alternative splicing which are described in the online database Universal Protein Resource (www.UniProt.org). These isoforms differ from the canonical one by the change or lack of several residues; the schematic representations of these structures are represented in Fig. (**1**). Isoform 2 is the most similar to the canonical, with a length of 1862 amino acids and domain conservation [53]. In the isoform 3 (1640 amino acids) the second polyserine tract and PHD domain are missing [56]. Isoform 4 is the shortest, containing only the first half of RAI1 protein (966 amino acids) [57] not encompassing NLSs and being comparable to the truncated protein found in some SMS patients. This short isoform has been found in muscle, while the other proteins have been described from brain tissue. In addition to the isoforms 2, 3 and 4 of RAI1, the online database of eukaryotic genomes, Ensembl project (www.ensembl.org) reports other two transcripts (ENST00000395776 and ENST00000428973), also generated by alternative splicing and both produce the same RAI1 protein of 1993 amino acids (ENSP00000379122). This protein shares a 100% homology with the isoform 1 until the residue 1855. It is important to note that in the same database, several transcripts of RAI1 were found, and these are consistent with the records of the other variants reported in the databases of UniProt and UCSC Genome Browser (http://genome.ucsc.edu/). 

Besides the domains described above for the protein, there are several post-translational modifications (PTM) sites found in RAI1, both *in silico* and experimentally. Some experimental evidence of PTM present in the RAI1 protein comes from *in vitro* studies where the isoform 1 and five RAI1 mutated forms of the protein (*RAI1-HA* 2687delC, *RAI1-HA* 3103delC, RAI1 R960X, RAI1-HA Q1562R, and RAI1-HA S1808N, four of them associated with SMS clinical phenotype) were studied. In this report all proteins presented a higher molecular weight than expected when run in an SDS-PAGE gel analysis. Moreover, the PTM were be mapped to the N-terminal of RAI1 [34]. Through the analysis of RAI1 sequence with NetGlycate 1.0, NetOGlyc 3.1 and YinOYang 1.2 servers [58-60], we found several predicted glycation sites homogenously located within the protein sequence and more interestingly, there are several O-glycosylation sites with high score, most of them mapping in the first amino-terminal half of RAI1. There are also O-linked β-N-acetylglucosamine (O-GlcNac) predicted sites with high score, which are present in many transcription factors and neuronal proteins; this modification participates in transcription, translation, proteasomal degradation and apoptosis among others and is directly involved in the regulation of neuronal development and synaptic transmission [61].

Phosphorylation is a well established PTM, implicated in protein regulation in a variety of cellular processes. We ran an *in silico* analysis of putative phosphorylation sites for RAI1 (NetPhos 2.0 Server [62]), and found the number of putative phosphorylable amino acid/total as follows: Ser128/217, Thr36/99, and Tyr13/39, indicating the existence of several possible phosphorylation sites. Consistent with these predictions, in recent studies to assess the global *in vivo* phosphoproteome one threonine (Thr1186) and thirteen serines (Ser568, 571, 574, 687, 1107, 1108, 1110, 1121, 1122, 1352, 1358, 1374 and 1616) were identified in a phosphorylated state in the RAI1 protein, and three of them (Ser568, Ser571 and Ser574) were regulated upon EGF stimulation in HeLa cells [63-66]. Interestingly, most of these sites are consensus for CK1, GSK3, NEK6 and ERK kinases. 

Lysine acetylation is a reversible PTM, which neutralizes the positive charge of this amino acid, changing protein function. This PTM is very important in the regulation of gene expression through the modification of histone proteins. Site-specific acetylation of a growing number of non-histone proteins has been shown to regulate their activity, localization, specific interactions as well as stability/degradation, therefore controlling a variety of cellular processes, such as transcription, proliferation, apoptosis and differentiation [67]. Interestingly, in another large scale analysis [68] an acetylation modification was reported for RAI1 at the peptide KGLEQGGKASDGISK in the Lysine 774 (underlined), which also presented a high accessibility rate in the 3D structure.

SUMOylation (Small Ubiquitin-like Modifier proteins) is a post-translational modification involved in various cellular processes, such as nuclear-cytosolic transport, transcriptional regulation, apoptosis, protein stability, response to stress, and progression through the cell cycle [69, 70]. *In silico* analysis (SUMOsp 2.0 [71]) of the RAI1 protein sequence gave seven putative SUMOylation sites of the TypeI: Ψ-K-X-E, in Lys549, 811, 819, 988, 1328, 1425 and 1500 (being Lys819 the most probable one). Therefore, the prediction of several sites for PTM within RAI1 sequence and the difference between the obtained vs. expected molecular weight of the protein, are consistent with a PTM in the N-terminal domain of RAI1 (Fig. (**1**)).

## RAI1 MOLECULAR AND CELLULAR FUNCTION

Little is known about the cellular and developmental role of RAI1. The first study was made in a mouse embryonic carcinoma cell line (P19) where the expression of GT1, a splice variant of Rai1 was markedly up-regulated after the treatment with retinoic acid for inducing neuronal differentiation [72]. 

Initially it was suggested, based in bioinformatic analyses that RAI1 could function as a transcriptional regulator [30, 31]. RAI1 and TCF20, a transcriptional cofactor, share a homology of 50% at the amino acid level, and both genes have a similar structure. Furthermore, the presence of the polyglutamine stretch at the N-terminal of RAI1 is coincident with many transcription factors containing polyglutamine activation domains and it has been shown that these stretches can modulate the transcription activation [73]. Consistent with this speculation, it was reported that mouse Rai1 has transactivational activity in HeLa cells but in moderate levels [48]. Considering it had milder activity than classical transcription factors, this can indicate that Rai1 might not act alone but may be part of a complex that regulates transcription. In 2010, an independent study showed that mouse Rai1 had much stronger transactivation activity in Neuro-2a cells (a neuronal derived cell line) than in HeLa cells [34]. These results suggest specificity in the transcription machinery that RAI1 could be part of and further indicate the importance of its role in neuronal derived tissues, since *RAI1* is a dosage sensitive gene involved in neurobehavioral phenotypes. Moreover, it was also demonstrated that human RAI1 has transcription factor activity [34] and is able to localize to the nucleus. Intriguingly, the two missense mutations (RAI1 Q1562R and RAI1 S1808N) were shown to localize to the nucleus and activate transcription of a reporter gene in the same magnitude than the wild type protein, strongly suggesting that these mutations are involved in other functions yet unknown for RAI1. The analysis of the mutations *RAI1-HA* 2687delC, *RAI1-HA* 3103delC and RAI1 R960X showed that they generated a truncated polypeptide with cytoplasmatic localization and higher transcription factor activity than the wild type protein. In addition, two different C-terminal halves of RAI1 protein (1038 aa-end and 1229 aa-end) were able to localize into the nucleus but had no transactivation activity. Taking all this into consideration plus the mouse protein studies previously reported [48], two functional domains were assigned to each half of RAI1: the N-terminal that contains transactivational activity and the C-terminal half of RAI1 responsible for the correct subcellular localization of the protein. In a normal situation the two functional domains of the protein are together. This suggests that the pathogenic outcome of some of the mutated forms may be explained by the dissociation of these two domains. However, in this context it is intriguing, and needs further study, the contribution of the different RAI1 isoforms to the final phenotype.

Therefore, it is clear that RAI1 is regulating gene expression and there are new data from cells and mice regarding to this aspect of the protein. A genome-wide gene expression study made in HEK293T (human embryonic kidney cells) with a 50% knockdown of *RAI1* generated by siRNA was performed using microarrays [74]. The top dysregulated genes found in these *RAI1* haploinsufficient cells are involved in neuronal differentiation, circadian activity and behavior; besides growth signaling, insulin sensitivity, gene expression, cell-cycle regulation, lipid biosynthesis, fat mobilization and skeletal, cardiovascular and renal development. The top candidate genes found upregulated due to *RAI1* haploinsufficiency are *ZIC1*, *PSEN2*, *RXRB*, *CLN8* and *SCN12A* being involved in neurogenesis, mental retardation, sensory transmission, nociceptive behavior, epilepsy and neurological function. Amongst the top candidate genes that are downregulated there are *NF1*, *MLL* and *NR1D2*, which are associated to schizophrenia, neurofibromatosis type I and circadian activity [74].

Recently in the mouse model for *Rai1* haploinsufficiency, *Rai1*^+/-^, several genes were reported to be dysregulated in the hypothalamus [75]. These genes include *Slc6a12* and *Slc38a5*, which are related to traits like epilepsy, anxiety disorder, mental retardation, whose expression is downregulated as a consequence of the haploinsufficiency of *Rai1*. Furthermore, it was found that human RAI1 isoform 1 directly regulates the expression of BDNF (brain-derived neurotrophic factor) [75] which is a neurotrophin that regulates neuronal survival, differentiation and growth during brain development, among others. The expression of this gene is known to be reduced in patients with Alzheimer, Parkinson and Huntington diseases [76]. Additionally, BNDF has been reported to be associated with obesity and hyperphagia [77, 78]. 

Moreover, altered gene expression was found for many genes in a hippocampus expression profile of the *Dup *mouse model for PTLS [15]. In this study the transcript of Ataxin 2, a protein mutated in spinocerebellar ataxia type 2 (SCA2), had a fold change of 1.24 by microarray analysis; certainly suggesting a link between its expression and the overexpression of* Rai1*. 

Altogether, the studies showing that CNV of *RAI1* causes dysregulation of many genes involved in many different cellular processes, suggest that RAI1 functions through specific pathways involved in the regulation of various biological processes, and when disrupted result in the phenotypic effects observed in SMS, PTLS and other neurobehavioral traits. 

## *RAI1* HAS BEEN IMPLICATED IN SEVERAL NEUROBEHAVIORAL TRAITS

As mentioned before, the haploinsufficiency of *RAI1* causes SMS meanwhile the duplication of a small region containing this gene is able to cause PTLS. Besides the strong association of RAI1 with these two genomic disorders, this protein has been reported to be related with other neurobehavioral traits. 

At the N-terminus of the RAI1 protein there is a polymorphic CAG repeat, which length is associated with the age of onset of spinocerebellar ataxia type 2 [79] and the response to neuroleptic medication in schizophrenia [80]. In addition, RAI1 has been shown to be associated with non syndromic autism [81]. 

Spinocerebellar ataxia type 2 (SCA2) is an autosomal dominant disease caused by the increment of a CAG repeat tract in ataxin-2 gene, generating an expanded polyglutamine tract in this protein. A study of the association between CAG repeat length and age at onset variability in SCA2 [79] in more than 100 patients found that *RAI1* locus contributes to 4.1% of the variance in SCA2 age at onset, after the effect caused by the expanded ataxin-2 protein. Therefore, this is the only association reporting RAI1 as a contributor in a neurodegenerative disorder, and due to a larger polyglutamine tract in RAI1 protein, which ranges from 10 to 16 glutamines in the normal population [33]. 

It was reported that a CAG repeat polymorphism in *RAI1* is associated with both the severity of the phenotype and the response to medication in schizophrenic patients [80]. Compared to non-responders, schizophrenic subjects responding to neuroleptic medication are characterized by a later age of onset and a better long-term outcome. The group responding to medication carried a significantly shorter CAG repeat size compared to control groups. 

It is remarkable to mention that CAG repeat polymorphism of *RAI1* have not been found to be causative of the SMS phenotype; and the length of this CAG repeat has not been analyzed in PTLS patients. The most common polyglutamine tracts in normal and SMS patients are 13-14 Gln. The comparison of phenotypes in SMS deletion patients with different number of CAG repeats did not show any association between CAG repeat length and the severity of SMS phenotype [33]. Recently, in a study with a group of patients with non-complex-autism, *RAI1* was found to be one of the novel autism candidate genes within the autism-associated CNVs regions [81]. In this study, *RAI1 *was the top most significant gene identified in the analysis of 173 non-overlapping CNV regions. 

All of these data indicate that RAI1 is participating in different aspects of behavior regulation. In Fig. (**2**) are summarized the neurobehavioral traits related with *RAI1* variations. In summary, there is plenty of evidence indicating that *RAI1* is the dosage sensitive gene responsible for most of the phenotypes of SMS and PTLS syndromes, including several neurobehavioral traits. 

It is also possible to suggest that RAI1 might have a role modulating some aspects of neuron function or differentiation since in addition to its contribution in both SMS and PTLS phenotypic outcome, it is highly expressed in neurons, its expression is influenced by retinoic acid and has polyglutamine polymorphisms that have been found to be associated with schizophrenia and spinocerebellar ataxia type 2. Taking into consideration the information known to date from studies *in vitro*, it can be hypothesized that at the cellular level the cause of the resulting phenotypes related to alterations in RAI1 can be due to (either or together) an abnormal translocation of the protein, reduced transactivational activity or inability of interaction with other proteins and/or DNA due to changes in a specific motif or to modifications in the 3D structure.

However, further studies are necessary with RAI1 and generally with all the other dosage sensitive genes known up to date in order to answer the main question that remains unanswered: what makes a gene, or its product, a dosage sensitive one?

## Figures and Tables

**Fig. (1):**
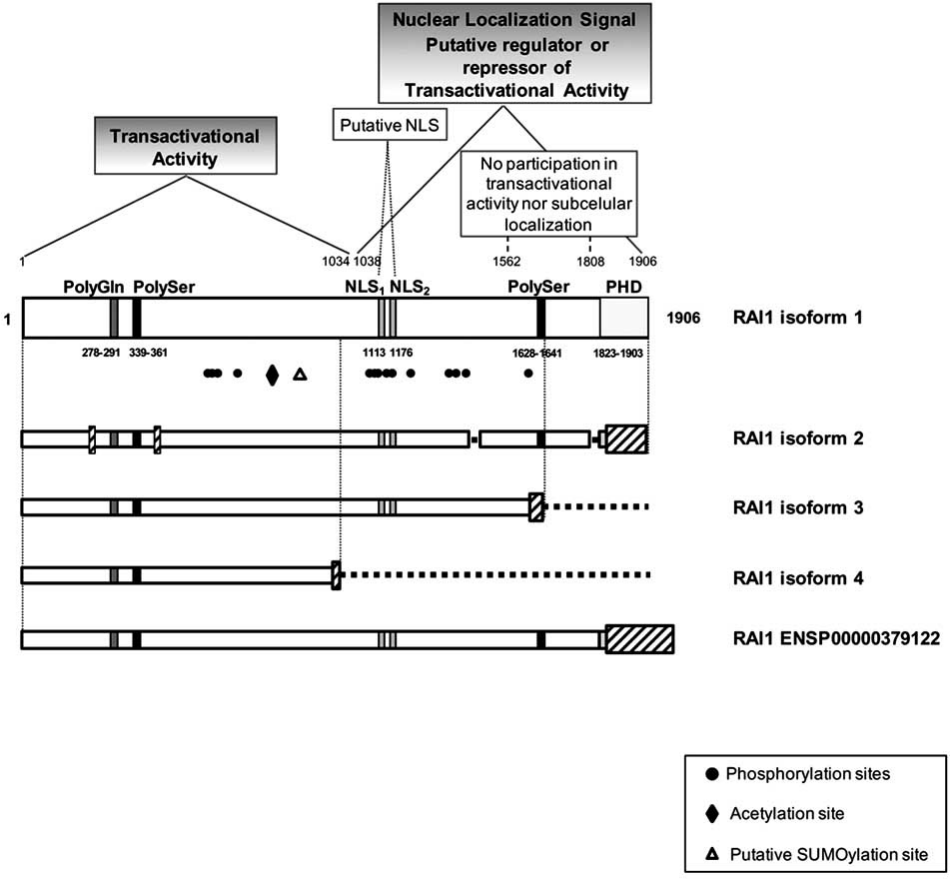
**Structure of RAI1.** By *in silico* analyses, several domains have been found for RAI1: a polyglutamine tract at the N-terminal of the protein, two polyserine domains, a PHD domain at the C-terminal of RAI1 and two putative nuclear localization signals (NLS). RAI1 protein structure is schematically represented, and the domains described are showed with their localization in the amino acidic sequence. By the evaluation of the wild type and mutant proteins associated to SMS, two main domains were found: the N-terminal half of RAI1 is responsible for the transactivational activity and C-terminal half beginning in residue 1038 is responsible for nuclear localization [34]. The RAI1 PTMs found are depicted with black figures while the putative sites are represented by white figures. The phosphorylation sites (1Thr and 13Ser) found in HeLa cells are represented with black circles. The reported acetylation site is depicted with a black diamond. The putative SUMOylation site with the highest score is represented with a white triangle. Below, the other four isoforms described for human RAI1 are represented. These isoforms differ from the canonical one by the change of several residues (depicted in slanted rectangles) and also by some fragments missed (depicted by discontinued lines). Isoform 2 is the most similar to the canonical, while in isoform 3 are missing the second polyserine tract and PHD domain. Isoform 4 is the shortest containing only the first half of RAI1 protein, not encompassing NLSs and being comparable to the truncated proteins found in some SMS patients. The longest variant of RAI1 (ENSP00000379122) is a protein reported by the online database of eukaryotic genomes, Ensembl, which has a length of 1993 amino acids and has complete homology with the isoform 1 until amino acid 1855.

**Fig. (2):**
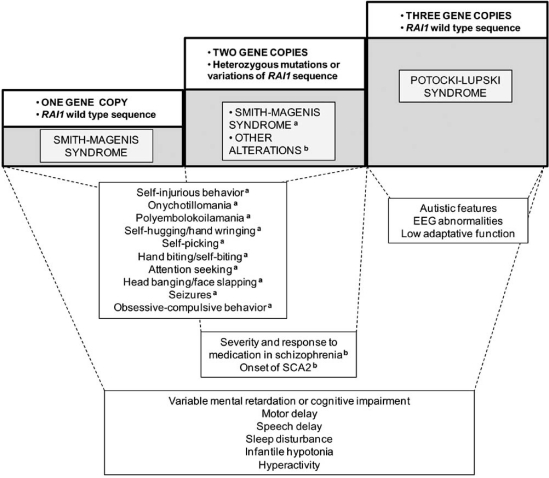
***RAI1* changes associated with neurobehavioral phenotypes.** The traits that have been found related to *RAI1* CNVs, mutations or sequence changes are summarized. There are phenotypes that are associated with only one copy of *RAI1* gene (SMS). Many of these phenotypes are also related to carrying two copies of the gene, but one allele with mutations (SMS caused by frameshifts, nonsense or missense mutations) ^(a)^. Harboring two copies of *RAI1* gene but with polymorphic CAG repeats influence other neurobehavioral traits ^(b)^. The alteration of *RAI1* dosage to three copies causes specific phenotypes of PTLS as well as features that overlap with some clinical characteristics of SMS.

**Table 1:** Key Neurobehavioral Features Found in SMS and PTLS Patients

Neurobehavioral features	SMS		PTLS	
	% in common deletion	% in RAI1 mutations	% in common duplication	% in small duplications
Variable mental retardation or cognitive impairment	100	100	100	100
Motor and speech delay	>90	70	100	100
Sleep disturbance	90	100	66.6	75
Infantile hypotonia	>90	61	88.8	75
Overt seizures	11–30	16.6	NR	NR
EEG abnormalities	49	NR	66.6-70	5.8
Attention seeking	80–100	100	NR	NR
Onychotillomania	25–85	100	NR	NR
Polyembolokoilamania	25–85	80	NR	NR
Hand biting/self-biting	80	60	NR	NR
Self-injurious behaviors	70–90	100	NR	NR
Self-hugging/hand wringing	50–80	100	NR	NR
Head banging/face slapping	70	90	NR	NR
Self-picking	54	100	NR	NR
Hyperactive	80	100	>70	66.6
Autistic features	NR	NR	87.5	66.6

A comparison between the phenotypes present in patients with the genomic rearrangements and point mutations of *RAI1* gene is shown. For SMS, the clinical features are presented in the percentage of patients carrying either the common 17p11.2 deletion (~3.7 Mb) or a mutation in *RAI1* gene. The percentages of features in PTLS are represented for patients that harbor the common 17p11.2 duplication (~3.7 Mb) or small duplications (<1 Mb). (NR: not reported) [25, 27-29].

**Table 2:** *RAI1* Gene Variations Related with Neurobehavioral Phenotypes

Mutation	Exon	Resultant protein	Mental retardation	Sleep disturbance	Speech delay	Motor delay	Self injurious behavior	Developmental delay	Hypotonia
^32^253del19	3	Missincorporation of 60 aa	+	+	+	+	+	NR	+
^35^1119delC	3	Missincorporation of 65 aa	+	+	NR	NR	+	NR	NR
^30^1449delC	3	Missincorporation of 34 aa	+	+	NR	+	+	NR	+
^30^2773del29	3	Missincorporation of 8 aa	+	+	+	NR	+	NR	NR
^31^C2878T	3	R960X	+	+	NR	NR	+	NR	NR
^31^3103insC	3	Missincorporation of 30 aa	+	+	NR	NR	+	NR	NR
^33^3103delC	3	Missincorporation of 28 aa	+	+	+	NR	+	NR	NR
^32^3801delC	3	Missincorporation of 46 aa	+	+	NR	+	+	NR	NR
^35^4649delC	3	Missincorporation of 36 aa	+	+	NR	NR	+	NR	NR
^32^A4685G	3	Q1562R	+	+	+	+	+	NR	+
^35^4933delGCCG	3	Missincorporation of 35 aa	+	+	NR	NR	+	+	NR
^30^5265delC	3	Missincorporation of 74 aa	+	+	NR	NR	+	NR	NR
^32^G5423A	3	S1808N	+	+	+	NR	+	NR	+
^33^A3634G ^33^(PolyQ)_18_	3 3	S1212G (PolyQ)_18_	+	>+	>+		>+	>+	>+

The mutations found in SMS patients are listed with their position in the cDNA, the protein that they generate and the neurobehavioral phenotypes associated with each mutation (+ means the presence of the characteristic, NR: not reported) [30-33, 35].

**Table 3:** *Rai1* CNV is Sufficient to Cause Most of the Phenotypes of SMS and PTLS also in Mice

Mouse model\Phenotype	*Del*	*Rai1^+/-^*	*Dup*	*Dup/Rai1^-^*	*Tg Rai1*
Overt seizures	Present (20%)	Subtle	No present	No present	No present
EEG	Abnormal	Abnormal	Normal	ND	ND
Locomotor activity	Hypoactive	Abnormal	Hyperactive	Hyperactive	Hyperactive
Anxiety	Decreased	Abnormal	Increased	Normal	Increased
Learning and memory	Normal	Normal	Impaired	Normal	ND
Social novelty recognition	Abnormal	ND	Impaired	ND	Abnormal
Dominance like behavior	Decreased	ND	Increased	ND	Increased
Reference	[16, 44, 45]	[48-50]	[15, 16, 44, 45, 50]	[50]	[51, 52]

Summary of the neurobehavioral phenotypes observed in existing mouse models for SMS and PTLS. *Del* and *Rai1^+/-^* mice carry one gene copy of *Rai1*, *Dup* model carries three copies, Tg *Rai1* has several copies and *Dup/Rai1^-^* has two copies or *Rai1* gene. (N/D: not determined).
